# Visualization of the Membranous Labyrinth and Nerve Fiber Pathways in Human and Animal Inner Ears Using MicroCT Imaging

**DOI:** 10.3389/fnins.2018.00501

**Published:** 2018-07-31

**Authors:** Rudolf Glueckert, Lejo Johnson Chacko, Dominik Schmidbauer, Thomas Potrusil, Elisabeth J. Pechriggl, Romed Hoermann, Erich Brenner, Alen Reka, Anneliese Schrott-Fischer, Stephan Handschuh

**Affiliations:** ^1^Department of Otolaryngology, Medical University of Innsbruck, Innsbruck, Austria; ^2^University Clinics Innsbruck, Tirol Kliniken, University Clinic for Ear, Nose and Throat Medicine Innsbruck, Innsbruck, Austria; ^3^Department of Biotechnology and Food Engineering, Management Center Innsbruck (MCI), Innsbruck, Austria; ^4^Department of Anatomy, Histology and Embryology, Division of Clinical and Functional Anatomy, Medical University of Innsbruck, Innsbruck, Austria; ^5^VetImaging, VetCore Facility for Research, University of Veterinary Medicine, Vienna, Austria

**Keywords:** microCT, inner ear implants, membranous labyrinth, non-destructive imaging, microanatomy, endolymph, perilymph, nerve fiber tracking

## Abstract

Design and implantation of bionic implants for restoring impaired hair cell function relies on accurate knowledge about the microanatomy and nerve fiber pathways of the human inner ear and its variation. Non-destructive isotropic imaging of soft tissues of the inner ear with lab-based microscopic X-ray computed tomography (microCT) offers high resolution but requires contrast enhancement using compounds with high X-ray attenuation. We evaluated different contrast enhancement techniques in mice, cat, and human temporal bones to differentially visualize the membranous labyrinth, sensory epithelia, and their innervating nerves together with the facial nerve and middle ear. Lugol’s iodine potassium iodine (I_2_KI) gave high soft tissue contrast in ossified specimens but failed to provide unambiguous identification of smaller nerve fiber bundles inside small bony canals. Fixation or post-fixation with osmium tetroxide followed by decalcification in EDTA provided superior contrast for nerve fibers and membranous structures. We processed 50 human temporal bones and acquired microCT scans with 15 μm voxel size. Subsequently we segmented sensorineural structures and the endolymphatic compartment for 3D representations to serve for morphometric variation analysis. We tested higher resolution image acquisition down to 3.0 μm voxel size in human and 0.5 μm in mice, which provided a unique level of detail and enabled us to visualize single neurons and hair cells in the mouse inner ear, which could offer an alternative quantitative analysis of cell numbers in smaller animals. Bigger ossified human temporal bones comprising the middle ear and mastoid bone can be contrasted with I_2_KI and imaged in toto at 25 μm voxel size. These data are suitable for surgical planning for electrode prototype placements. A preliminary assessment of geometric changes through tissue processing resulted in 1.6% volume increase caused during decalcification by EDTA and 0.5% volume increase caused by partial dehydration to 70% ethanol, which proved to be the best mounting medium for microCT image acquisition.

## Introduction

Cochlear implant electrodes are nowadays offered individually tailored to recipient’s needs, as there is a huge anatomical variation of the cochlea length ranging from 28 to 42 mm at the level of the sensory epithelium from base to apex (Hardy, 1938; Spoendlin and Schrott, 1989; Wysocki, 1999; Escude et al., 2006; Erixon et al., 2009; Biedron et al., 2010; Rask-Andersen et al., 2011; Johnson et al., 2014). Newly designed electrodes for a future vestibular implant aim to restore vestibular function after bilateral loss that cannot be compensated by other sensory inputs. This raises the need for a thorough morphometric evaluation of variations in anatomical structures of the inner ear. The premise for vestibular implantation is to leave the membranous labyrinth as intact as possible in order not to impair hearing function by penetration of the endolymphatic compartment. On the other hand, stimulating electric contacts shall be placed as close as possible to sensorineural structures to limit unwanted current spread and cross excitation of neighboring nerves. Hence, there is a need to analyze the endolymphatic compartment and nerve fiber pathways within the bony labyrinth in a statistically representative number of human inner ears to assess anatomical variation with high level of detail. Conventional clinical tools such as magnetic resonance imaging (MRI) and X-ray computed tomography (CT) offer only limited contrast and resolution of soft tissues within the temporal bone. Clinical 7 T high-field MRI has been used to image the inner ear *in vivo* at 0.3 mm isotropic voxel resolution (van der Jagt et al., 2015). While 7 T MRI allows depicting large features such as nerves or semi-circular canals (van Egmond et al., 2014), ultra high-field MRI at 11.7 T of *ex vivo* cadaveric temporal bones at 50 μm isotropic voxel resolution even allows discriminating delicate features such as Reissner’s membrane and the all scala with high contrast (Thylur et al., 2017), thus providing valuable data for rapid segmentation of perilymphatic and endolymphatic compartments. Still, ultra high-field MRI does not provide sufficient spatial resolution for imaging of microscopic features of the inner ear such as thin nerve fiber bundles. Thus it is necessary to image the inner ear by microscopic imaging modalities. Three-dimensional visualization by reconstruction of histological serial sections is very laborious and implies limited in *z*-resolution, which degrades overall resolution (Biedron et al., 2010) but provides highest level of cellular detail in the *x*–*y* cutting plane. Non-destructive 3D imaging with conventional microscopic computed tomography (microCT) offers isometric accurate quantitative morphology for studying the complex mammalian inner ear down to a resolution of few micrometers at reasonable costs and high throughput. Established for visualization of mineralized tissue, the use of hard X-rays requires some contrast enhancement of soft tissue since the X-ray attenuation coefficient of inner ear fluids and membranous soft tissue structures are highly similar (Avci et al., 2014; Elfarnawany et al., 2017). New phase-contrast imaging with polychromatic X-rays uses, besides the local object absorption, X-ray refraction, and scattering in object features to increase contrasts. Nevertheless, the inner ear presents a considerable challenge for all kind of X-ray imaging techniques, since it is situated within the bone with one of highest mineral densities in the mammalian body (high X-ray absorption) and contains delicate membranous structures (very low X-ray absorption).

We tested several contrast enhancement agents to selectively display the membranous labyrinth, nerve fiber bundles and soft tissue in human and animal inner ears before and after decalcification of mineral components. Assessment of some tissue changes due to preparation procedures and 3D reconstructions of segmented structures were performed. Focus of this work was laid to selectively visualize main fluid compartments together with nerve bundle pathways of all sensory structures in the inner ear. This shall allow engineers and surgeons to find best ways to access and stimulate malfunctional sensory structures. The data from 52 human temporal bones shall provide a basis for a morphometric analysis of the variability of human inner ear anatomy (Johnson Chacko et al., 2018) and simulation of current spread with finite element analysis (Handler et al., 2017).

## Materials and Methods

Specimens prepared for the present study included adult C57 Bl6/N mice (30–60 days old), adult cats (10 month), and human inner ear samples from subjects aged from 6 to 90 years without any known hearing or balance disorders. A summary of sample preparation steps and tomography acquisition parameters is given in **Table 1**. Detailed sample preparation and imaging protocols including exact information on chemical reagents and sample processing times and conditions are provided in Supplementary Data Sheet S1. Detailed information on image processing and analysis is provided in Supplementary Data Sheet S2.

**Table 1:** Overview on specimen preparation and image acquisition parameters for all specimens shown in this paper.

Sample information		Sample preparation				Tomography parameters				
		
Species	Sample	Fixative	Stain	Decalcified	Scanning medium	Scanner	kVp	μA	Exposure (s)	Voxel size (μm)
Mouse	Inner ear	4% PFA	I_2_KI	No	Distilled water	Scanco μCT35	70	114	2	3.50
Mouse	Inner ear	4% PFA	I_2_E	No	100% Ethanol	Scanco μCT35	70	114	2	3.50
Mouse	Inner ear	4% PFA	Gastrografin	No	PBS	Scanco μCT35	70	114	2	3.50
Mouse	Inner ear	4% PFA	PTA	No	70% Ethanol	Scanco μCT35	70	114	2	3.50
Mouse	Inner ear	4% PFA	OsO_4_	No	PBS	Scanco μCT35	70	114	2	3.50
Mouse	Inner ear	Karnovsky	OsO_4_	Yes	Epoxy	Scanco μCT35	45	177	2	3.50
Mouse	Inner ear	OsO_4_	OsO_4_	No	PBS	Xradia MicroXCT-400	70	114	30	2.19
Mouse	Inner ear	Karnovsky	OsO_4_	No	PBS	Xradia MicroXCT-400	70	114	30	2.19
Mouse	Inner ear	OsO_4_	OsO_4_	Yes	PBS	Xradia MicroXCT-400	45	110	30	2.19
Mouse	Inner ear	OsO_4_	OsO_4_	Yes	PBS	Xradia MicroXCT-400	40	75	360	0.49
Mouse	Inner ear	Karnovsky	OsO_4_	Yes	PBS	Xradia MicroXCT-400	45	110	30	2.19
Cat	Inner ear	4% PFA	–	No	Distilled water	Scanco μCT35	70	114	8	10.00
Cat	Inner ear	4% PFA	I_2_KI	No	Distilled water	Scanco μCT35	70	114	6.4	10.00
Human	Inner ear	Karnovsky	OsO_4_	No	PBS	Scanco μCT35	70	114	1.6	15.00
Human	Inner ear	Karnovsky	OsO_4_	No	PBS	Scanco μCT35	70	114	1.6	10.00
Human	Inner ear	Karnovsky	OsO_4_	No	Epoxy	Scanco μCT 100	70	182	1.6	3.00
Human	Inner ear	4% PFA	I_2_KI	No	Distilled water	Scanco μCT35	70	114	1.6	15.00
Human	Middle and inner ear	4% PFA	I_2_KI	No	Distilled water	Xradia MicroXCT-400	130	60	30	25.38
Human	Inner ear	Karnovsky	OsO_4_	Yes	70% Ethanol	Xradia MicroXCT-400	45	109	30	15.00
Human	Inner ear	Karnovsky	OsO_4_	Yes	70% Ethanol	Xradia MicroXCT-400	45	109	30	10.00
Human	Inner ear	Karnovsky	OsO_4_	Yes	70% Ethanol	Xradia MicroXCT-400	45	133	60	5.54


### Ethics Statement

Human bodies were donated to the Division of Clinical and Functional Anatomy of the Innsbruck Medical University by people who had given their informed consent prior to death for the use of their bodies for scientific and educational purposes (McHanwell et al., 2008; Riederer et al., 2012). All specimens were anonymized. There was no evidence for any malformation in any human temporal bone. All procedures for cat animal tissue were approved by the BRC located at Royal Victorian Eye and Ear Hospital and Animal Research and Ethics Committee, East Melbourne, VIC, Australia. Mice breeding and care were performed at the central animal facility in Innsbruck, Austria and experiments were approved by the Austrian Ministry of Science and Research and conformed to the Austrian guidelines on animal welfare and experimentation (BMWFW-66.011/0120-WF/V/3b/2016).

### Sample Preparation and Tomographic Image Acquisition

#### Mice

We tested the properties of five different contrast agents for staining soft tissue structures of the inner ear. All five contrast agents have previously been used for either staining soft tissues for microCT imaging or clinical radiography. Lugol’s iodine potassium iodine (I_2_KI) and elemental iodine in absolute ethanol (I_2_E) have been used for contrast enhancement in vertebrate soft tissue samples (Metscher, 2009a,b; Gignac and Kley, 2014; Gignac et al., 2016; Handschuh et al., 2017). Gastrografin^®^ is a water-based iodine compound with a long tradition of use in clinical radiography and CT (Lessman and Lilienfeld, 1959; Hong et al., 2010). Phosphotungstic acid (PTA) has been used for different vertebrate samples (Metscher, 2009a; Das Neves Borges et al., 2014) including studies on inner ear morphology in teleost fish (Schulz-Mirbach et al., 2013). Osmium tetroxide (OsO_4_) is the most commonly used chemical for post-fixation in electron microscopy tissue preparation and has already been utilized frequently as a contrast agent for microCT imaging (Johnson et al., 2006; Metscher, 2009b; Handschuh et al., 2013). Inner ears of 5 week old male C57BL/6N mice were perfused with 4% formaldehyde. washed in PBS, and inner ears incubated either with I_2_KI, I_2_E, Gastrografin^®^, PTA, or OsO_4_. After incubation, samples were washed and mounted in plastic sample holders for scanning. In addition, another OsO_4_-stained sample was decalcified and embedded in Epon. Samples were scanned using a Scanco μCT35 (SCANCO Medical AG, Brüttisellen, CH) with an isotropic voxel resolution of 3.5 μm.

In a second experiment we tested the specific properties of OsO_4_-staining based on two different fixation regimes. Two 40 days old C57BL/6N mice were fixed in Karnovsky’s solution and subsequently post-fixed with 2% OsO_4_, while two samples were directly fixed in 2% OsO_4_. For each fixation regime one sample was scanned without previous decalcification and the other sample was scanned after decalcification with EDTA. Decalcification was performed at neutral pH (pH 7.2–7.4) with EDTA in PBS for 270 min at 37°C with microwave support and magnetic stirring in a Milestone Histos 5 tissue processor. The four samples were washed and mounted in PBS and scanned with an XRadia MicroXCT-400 (Carl Zeiss X-ray Microscopy, Pleasanton, CA, United States) with an isotropic voxel size of 2.19 μm. In addition, a high-resolution interior tomography of the organ of Corti of the decalcified OsO_4_-fixed specimen was done with an isotropic voxel resolution of 0.49 μm.

#### Cat

The bony labyrinth of a 10 month old cat was excised, fixed in neutral buffered formaldehyde (4 %) and washed in PBS. A scan of the ossified bone specimen was acquired using a Scanco μCT35 with an isotropic voxel size of 10 μm. Subsequently, the sample was stained with I_2_KI washed and scanned with a Scanco μCT35 with an isotropic voxel size of 10 μm.

#### Humans

Fifty-two temporal bones from body donors were excised and fixed in Karnovsky’s solution for several weeks. To ensure rapid fixative penetration, oval and round windows were penetrated with a needle and the fixative gently perfused with a Pasteur pipette. Post-mortem time until fixation reached from 4 to 12 h. 48 of those specimens were post-fixed in OsO_4_. After thorough washes in PBS excess bone was removed with a drill to meet maximum specimen size for the microCT scanner. Scans from the ossified specimens were acquired using a Scanco μCT35 at 70 kVp with an isotropic voxel size of 15 μm. Subsequently specimens were decalcified in EDTA washed in PBS and mildly dehydrated to 70% ethanol to remove air bubbles present in PBS. Scans from the decalcified specimens were acquired using an XRadia MicroXCT-400 at 45 kVp with an isotropic voxel size of 15 μm. For evaluating the impact of voxel size on resolvable image features, one decalcified sample was also scanned at isotropic voxel sizes of 10 and 5.5 μm. For comparison, the remaining four specimens were stained with I_2_KI solution. Two specimens were drilled to the typical sample diameter and scanned using a Scanco μCT35 with an voxel size of 15 μm. The other two specimens were left larger in order to visualize middle ear structures together with the inner ear without any decalcification. Fixation and contrast agents could penetrate mainly via the Eustachian tube and inner ear canal. After incubation in I_2_KI the bone was imaged using an XRadia MicroXCT-400 at 130 kV with an isotropic voxel size of 25.38 μm.

One human specimen was fixed in Karnovsky’s mixture, post-fixed with OsO_4_ and embedded in Spurr’s epoxy resin (Spurr, 1969) without any prior decalcification. The cochlea was separated from the vestibule with a fretsaw and the block grinded to 100 mm × 50 mm× 50 mm. The plastic block was imaged using an ultra-high-resolution SCANCO^®^ VivaCT 100 microCT at Scanco Medical AG headquarter (Brüttisellen, Switzerland) with an isotropic spatial resolution of 3 μm.

### Image Processing, Visualization, and Analysis

#### Mice

**FIGURE 1:**
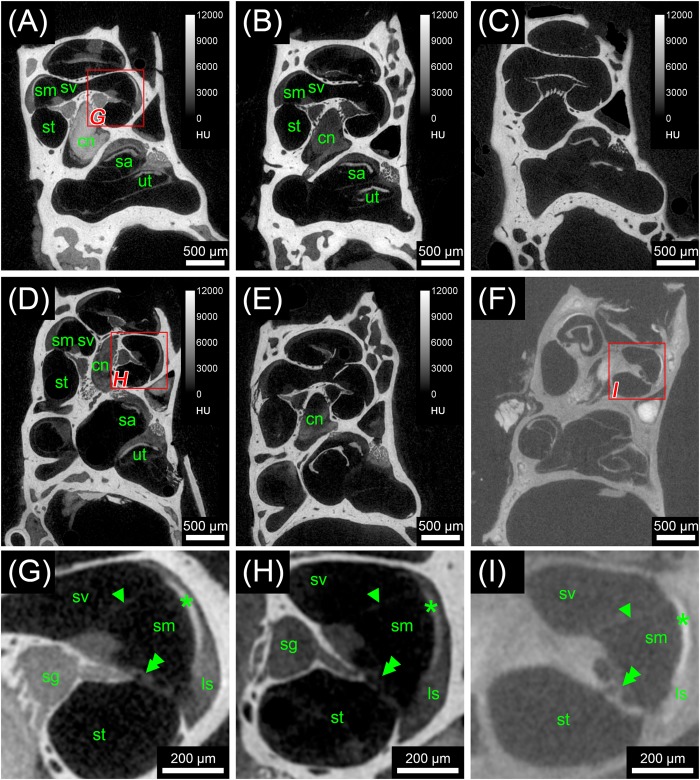
First staining experiment evaluating the potential of five different X-ray dense contrast agents for staining soft tissues of the inner ear in the mouse. For all specimens, the apex of the cochlea was opened to ensure good fixation and penetration of contrast agents. Voxel size for all scans was 3.50 μm isotropic. **(A)** Longitudinal virtual section through mouse cochlea stained with I_2_KI scaled to HU. **(B)** Longitudinal virtual section through mouse cochlea stained with I_2_E scaled to HU. **(C)** Longitudinal virtual section through mouse cochlea stained with Gastrografin^®^ scaled to HU. **(D)** Longitudinal virtual section through mouse cochlea stained with PTA scaled to HU. **(E)** Longitudinal virtual section through mouse cochlea stained with OsO_4_ scaled to HU. **(F)** Longitudinal virtual section through mouse cochlea stained with OsO_4_ and subsequently decalcified and embedded in epoxy resin. **(G)** High-magnification image of representative soft tissues of the I_2_KI-stained sample. **(H)** High-magnification image of representative soft tissues of the PTA-stained sample. **(I)** High-magnification image of representative soft tissues of the decalcified and resin-embedded OsO_4_-stained sample. Highest contrast for soft tissues is provided by I_2_KI **(A,G)**, PTA **(D,H)**, and OsO_4_ after decalcification and resin-embedding **(F,I)**, clearly depicting nerves, membranes, and bone marrow. In the cochlea, the sensory epithelium, spiral limbus as well as spiral ligament are clearly visible and the stria vascularis can be separated from the spiral ligament based on a higher staining intensity. Even delicate structures like Reissner’s membrane can be distinguished. Borders of the membranous labyrinth are visible in the cochlea, saccule, utricle, and semi-circular canals. I_2_E **(B)** and OsO_4_ **(E)** provide lower contrast, which made it more difficult to display bone and soft tissue within the same acquisition, although in case of OsO_4_ this related to problems with fixation or staining of the samples, as all other OsO_4_-treated specimens provided excellent image contrast (panel **F** and **Figure 3**). Gastrografin **(C)** failed to deliver a measureable increase in soft tissue contrast. Legend: ls, spiral ligament (*ligamentum spirale*); cn, cochlear nerve; sa, saccule; sg, spiral ganglion; sm, scala media; st, scala tympani; sv, scala vestibule; ut, utricle. Arrowheads, Reissner’s membrane; double arrowheads, organ of Corti; asterisk, stria vascularis; HU, Hounsfield units.

**FIGURE 2:**
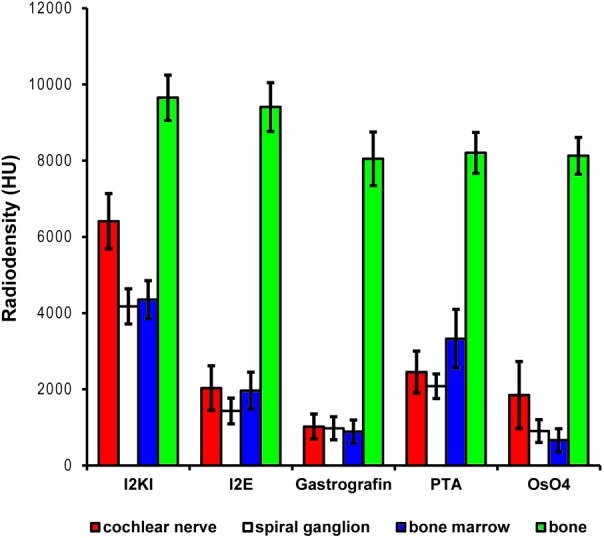
Quantitative evaluation of tissue contrast based on staining with five different contrast agents. Highest tissue contrast was achieved with I_2_KI staining for all measured soft tissues. PTA and I_2_E also yielded reasonable X-ray densities, while Gastrografin failed to deliver a measureable increase in soft tissue contrast. Voxel intensities in the OsO_4_-stained specimen were also very low but partly heterogeneous. In this sample, only outer parts of the cochlear nerve did bind some stain, while no contrast increase was observed in inner parts of the nerve. This is reflected by the higher standard deviation and most likely relates to technical problems during fixation and/or staining. Highest voxel intensities up to roughly 10,000 HU were observed in bone. Measured intensities in bone were highly similar for the Gastrografin, PTA, and OsO_4_ specimen. Slightly higher intensities measured in bone in the I_2_KI and I_2_E specimens are most likely relate to iodine uptake in bone. HU, Hounsfield units.

**FIGURE 3:**
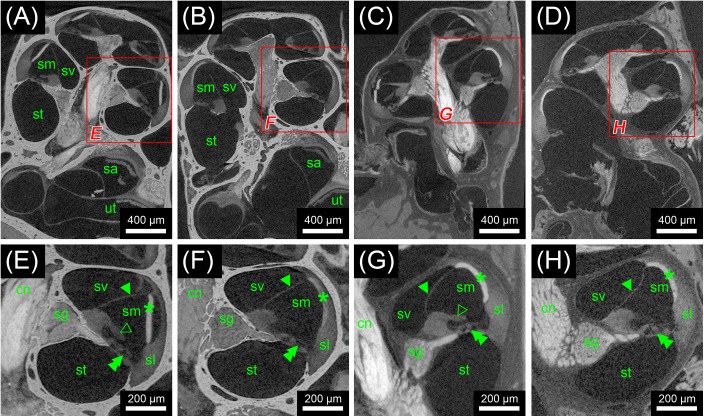
Second staining experiment evaluating the potential of OsO4 in ossified and decalcified specimens with and without prior aldehyde fixation in the mouse. Voxel size for all scans was 2.19 μm isotropic. **(A)** Longitudinal virtual section through an ossified mouse cochlea fixed with OsO_4_. **(B)** Longitudinal virtual section through an ossified mouse cochlea fixed with Karnovsky’s fixative and post-fixed with OsO_4_. **(C)** Longitudinal virtual section through a decalcified mouse cochlea fixed with OsO_4_. **(D)** Longitudinal virtual section through a decalcified mouse cochlea fixed with Karnovsky’s fixative and post-fixed with OsO_4_. **(E)** High-magnification image of representative soft tissues of the ossified OsO_4_-fixed sample. **(F)** High-magnification image of representative soft tissues of the ossified Karnovsky-fixed and OsO_4_-post-fixed sample. **(G)** High-magnification image of representative soft tissues of the decalcified OsO_4_-fixed sample. **(H)** High-magnification image of representative soft tissues of the decalcified Karnovsky-fixed and OsO_4_-post-fixed sample. In general, the OsO_4_ yielded excellent soft tissue contrast for all four specimens, although slight differences between the fixation regimes could be seen. In the OsO_4_-fixed specimens the stria vascularis was largely detached from the spiral ligament, which most likely represents a fixation artifact as this condition was not observed in the Karnovsky-fixed specimens. On the other hand, the tectorial membrane was only visible in the OsO_4_-fixed specimens! Legend: sl, spiral ligament; cn, cochlear nerve; sa, saccule; sg, spiral ganglion; sm, scala media; st, scala tympani; sv, scala vestibuli; ut, utricle. Arrowheads, Reissner’s membrane; open arrowheads, tectorial membrane; double arrowheads, organ of Corti; asterisk, stria vascularis.

Scans from the first staining experiment were imported to the software package Amira^®^ 6.2 (Thermo Fisher Scientific–FEI Visualization Sciences Group, Mérignac Cédex, France), converted to Hounsfield units (HUs) and filtered to reduce image noise. Virtual sections were inspected for image contrast and visibility of selected soft tissue structures of the inner ear (**Figure 1**). In addition, X-ray densities for different tissues including cochlear nerve, spiral ganglion, bone marrow, and bone were measured to quantify the staining for different contrast agents. Average voxel intensities including standard deviations are given in **Figure 2**. Scans from the second staining experiment (OsO_4_-staining based on different fixation regimes) were again inspected for image contrast and visibility of selected soft tissue structures of the inner ear (**Figure 3**).

#### Cat

**FIGURE 4:**
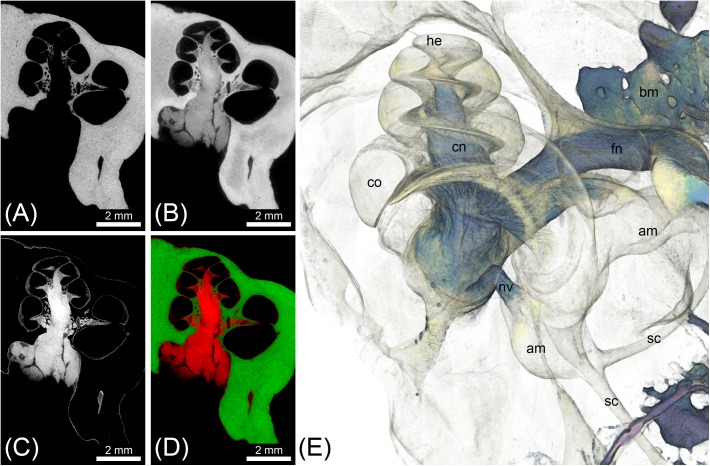
MicroCT imaging of a cat inner ear for visualization of mineralized and soft tissues. Voxel size was 10 μm isotropic. **(A)** MicroCT scan of the unstained cat inner ear specimen depicting the bony labyrinth. **(B)** MicroCT scan of the I_2_KI-stained cat inner ear specimen depicting the bony labyrinth along with big nerves (vestibular and cochlear nerve) and connective tissue such as the spiral ligament. **(C)** Image as a result from subtraction of a binary bone mask [threshold segmentation of **(A)**] from **(B)**, representing all stained soft tissues. **(D)** Overlay of [**(A)** green] and [**(C)** red]. **(E)** Volume rendering from the soft tissue volume **(C)**, revealing nerves, bone marrow, and the membranous labyrinth. Legend: am, ampulla; bm, bone marrow; co, cochlea; he, helicotrema; nc, cochlear nerve; nf, facial nerve; nv, vestibular nerve; sc, semi-circular canal.

The two scans from the cat specimen acquired before and after I_2_KI staining, were imported into Amira^®^ 5.5 and registered. From the unstained specimen, a binary segmentation mask was created based on threshold segmentation. Subsequently, this mask was subtracted from the I_2_KI-stained scan, which allowed selective visualization of soft tissue components (**Figure 4**).

#### Humans

**FIGURE 5:**
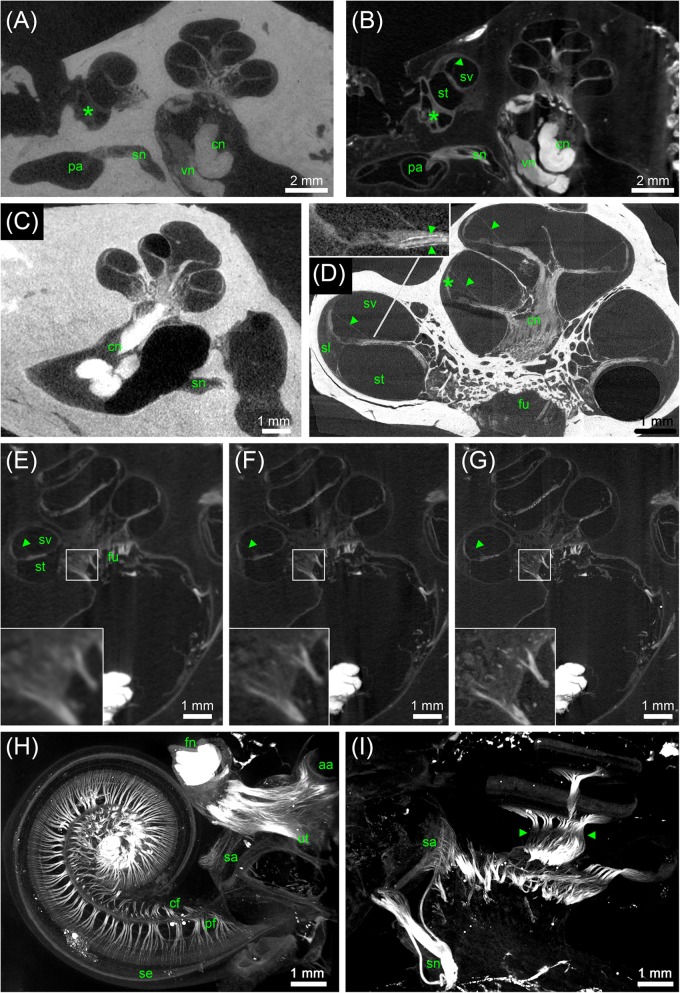
MicroCT of human inner ears: different preparations and voxel resolutions. **(A)** Midmodiolar view at the level of the round window (asterisk) of an ossified OsO_4_-post-fixed human temporal bone presenting the bony structure and low contrast of main nerve trunks of the cochlear (cn) and vestibular nerve (vn) in the inner ear canal. The singular nerve (sn) innervating the posterior ampulla (pa) lightly accentuated from the fluid filled bony canal; 15 μm voxel size. **(B)** Registered dataset of the same specimen after decalcification demonstrating the increased contrast of soft tissue. Note the fibrous connective tissue at the round window (asterisk)! The membranous labyrinth is clearly silhouetted against the perilymphatic compartment, Reissner’s membrane (arrowhead) allowed to clearly identify scala vestibuli (sv) and scala tympani (st); 15 μm voxel size. **(C)** I_2_KI contrasted ossified temporal bone depicts highest contrast in the central cochlear nerve (cn) but impeded to outline most other soft tissues; 15 μm voxel size. **(D)** 3 μm voxel sized scan of an ossified OsO_4_-post-fixed human inner ear embedded in epoxy resin. Reissner’s membrane (arrowheads) revealed high contrast, such as the stria vascularis (asterisk) and cochlear nerve (cn). Inset figure shows a magnified view of the sensory epithelium of the basal turn. The delicate osseous spirallamina lamelle (arrowheads) are clearly visible. **(E–G)** Registered datasets imaged at different voxel resolutions [**(E)** 15 μm, **(F)** 10 μm, and **(G)** 5.5 μm]. Insets show magnified views of cochlear nerve fibers traveling through the fundus region (fu) and display the increase of the level of detail. **(H,I)** Maximum intensity projection of sub volumes of the dataset shown in **(G)**; 5.5 μm voxel size. **(H)** Horizontal view of cochlear basal and middle turn displays the ramification of the peripheral nerve fiber bundles (pf) spreading towards the sensory epithelium (se). Central fibers (cf) coalesce to the cochlear nerve; facial nerve (fn) shows highest contrast. Single bundles of the nerve fiber meshwork extend into the vestibular end organs of the utricle (ut) and anterior ampulla (aa). **(I)** View perpendicular to the modiolar plane illustrates nerve bundles of the singular nerve and innervation of the macula sacculi (sa). The spiral nerve coil in the cochlea reveals darker areas (arrowheads) that correspond to unmyelinated somata of the spiral ganglion. Legends: aa, anterior ampulla; cn, cochlear nerve; fn, facial nerve; fu, fundus region; pa, posterior ampulla; pf, peripheral nerve fibers; sa, macula sacculi; se, sensory epithelium; sl, spiral ligament; sn, singular nerve; st, scala tympani; sv, scala vestibule; ut, utricle; vn, vestibular nerve.

For comparison of scans made from ossified and decalcified human inner ear specimens at identical voxel resolutions (15 μm), two scans from the same OsO_4_-stained specimen before and after decalcification were imported into Amira^®^ 6.2 and filtered for noise reduction. Subsequently, the two image volumes were registered and corresponding slices extracted to assess image contrast (**Figures 5A,B**). Results from this OsO_4_-stained specimen were compared to representative slices from an I_2_KI-stained inner ear scanned also at 15 μm voxel resolution (**Figure 5C**), as well as to the OsO_4_-stained specimen that was embedded in epoxy resin and scanned at 3 μm voxel resolution (**Figure 5D**). In order to assess the impact of voxel resolution on smallest detectable feature size in an OsO_4_-stained and decalcified specimen, three scans of the same specimen (15, 10, and 5.5 μm voxel resolution, respectively) were imported to Amira^®^ 6.2, filtered and registered. Corresponding slices were extracted to assess image resolution (**Figures 5E–G**). For illustrating smallest detectable nerve fibers in the cochlea, maximum intensity projection from thick slices were made using Amira^®^ 5.3.3 (**Figures 5H,I**).

### High-Resolution Sub-volume Imaging (Interior Tomography) of the Mouse Cochlea for Detecting Single Cells in the Cochlea

**FIGURE 6:**
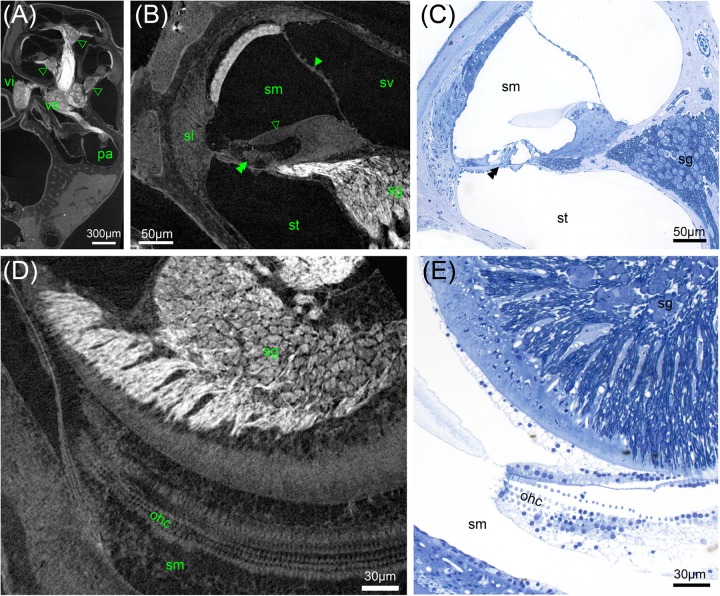
High-resolution microCT of a decalcified mouse inner ear: Solely OsO_4_-fixation results in excellent contrast of soft tissue **(A,B,D)**. **(A)** Myelinated nerve fiber areas contrast to lower X-ray densities in the spiral ganglion areas (arrowheads) and ganglion of the superior vestibular ganglion (vs) and inferior branch (vi) of the vestibular ganglion, 2.19 μm voxel resolution. **(B)** Sub-volume scan of **(A)** with 0.49 μm voxel size displays cellular resolution with bulging nuclei of the mesothelial layer of Reissner’s membrane (arrowhead). The acellular tectorial membrane (open arrowhead) clearly accentuates against the scala media (sm), even the three rows of outer hair cells can be resolved (double arrowhead), spiral ganglion neuron (sg) somata can be quantified. **(C)** A 1 μm thick semi-thin section emphasizes the high level of detail possible. **(D)** Horizontal sections may be ideal for neuron counting and outer hair cell (ohc) quantification. **(E)** Semi-thin section shows corresponding region of **(D)** with histological techniques. Legends: ohc, outer hair cell; pa, posterior ampulla; sg, spiral ganglion; sl, spiral ligament; sm, scala media; st, scala tympani; sv, scala vestibuli; vi, inferior vestibular ganglion; vs, superior vestibular ganglion.

The high-resolution scans of the mouse cochlea were filtered and inspected for detecting smallest resolvable image features in the inner ear. Virtual slices were compared to 1 μm thick semi-thin plastic sections stained with toluidine blue from corresponding regions of a different mouse inner ear specimen (**Figure 6**).

### Data Segmentation and 3D Visualization

**FIGURE 7:**
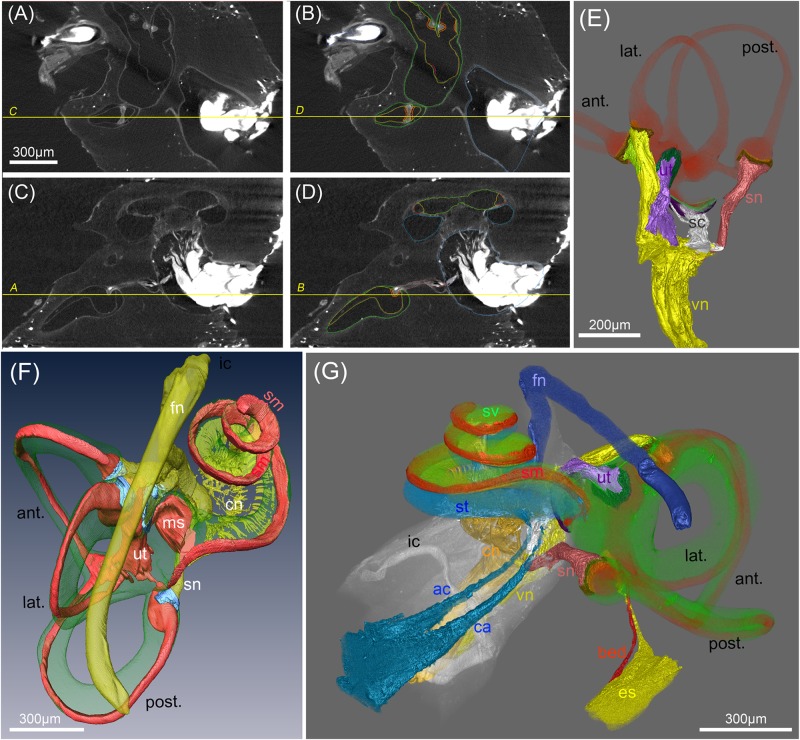
Segmentation of soft tissue structures in the human inner ears. **(A–D)** MicroCT views acquired at 15 μm voxel resolution before **(A,C)** and after **(B,C)** manual segmentation of soft tissue structures. Yellow lines and labeling represent orthogonal planes of corresponding figures to demonstrate high accuracy of segmentation in multiple dimensions. **(E)** Volume renderings of the peripheral vestibular nerves, sensory epithelia (orange) and membranous labyrinth (red). “Gap” regions between sensory epithelia and nerves are colored in brown **(F)** 3D surface rendering of vestibular and cochlear nerve (cn) (yellow), membranous labyrinth (red), ampullar vestibular sensory epithelia (light blue)). Facial nerve (fn) is colored yellow. **(G)** Volume rendering of a fully segmented human temporal bone with the cochlear aqueduct (turquoise) and a parallel accessory canal containing the inferior cochlear vein. Both emerged from the scala tympani (turquoise). The endolymphatic duct (yellow) widens into the endolymphatic sac that is not present in toto in this preparation. A putative blood vessel parallels the endolymphatic duct (red); membranous labyrinth red, perilymph (green). Legends: ac, accessory canal of the cochlear aqueduct; ant., anterior semi-circular canal; bed blood vessel endolymphatic duct; ca, cochlear aqueduct; cn, cochlear nerve; es, endolymphatic sac (intraosseous part); fn, facial nerve; ic, inner ear canal; lat., lateral semi-circular canal; ms, macula sacculi; post., posterior semi-circular canal; sc, saccular nerve; sm, scala media; sn, singular nerve; st, scala tympani; sv, scala vestibuli; ut, utricular nerve; vn, vestibular nerve.

**FIGURE 8:**
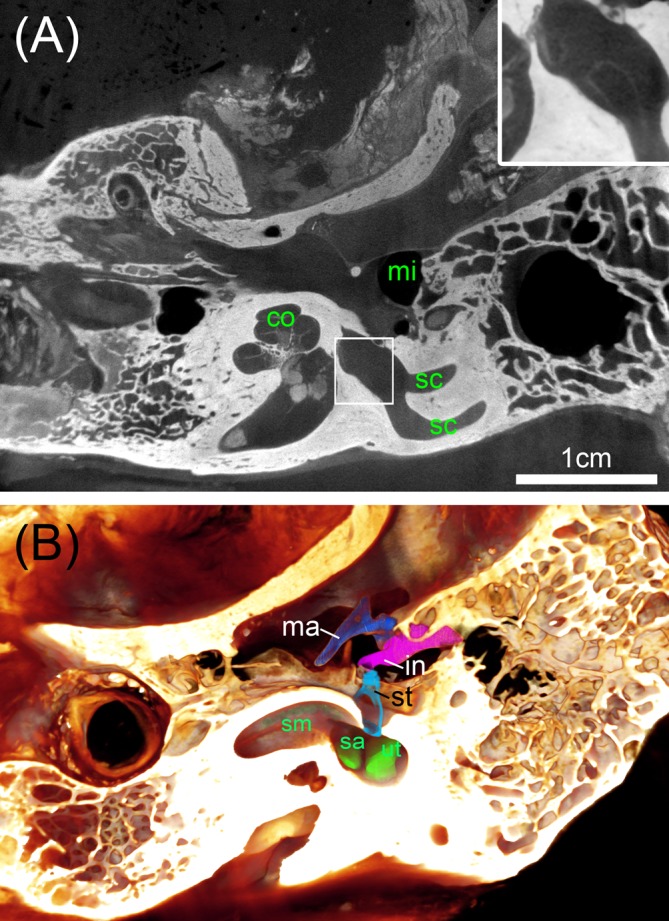
Big ossified human temporal bones with iodine staining. **(A)** 25 μm voxel sized scan identifies the bone distinct from soft tissue with sufficient contrast to outline even bigger parts of the membranous labyrinth shown in the high magnified inset. **(B)** Volume rendering is suitable to assess the extent of the membranous labyrinth of the scala media (sm) and endolymphatic compartments of the sac (sa) and utricle (ut) close to the oval window that is occupied by the stapes (st). Legend: co, cochlea; in, incus; ma, malleus; mi, middle ear; sc, semi-circular canal; st, stapes.

As part of a project that addressed mean shape and shape variability in soft tissues focusing on the vestibular system (Johnson Chacko et al., 2018; Johnson Chacko et al., 2018), 50 scans acquired at 15 μm voxel size were imported to Amira^®^ 6.2 and nerves and structures of the membranous labyrinth were manually segmented switching between the 3 orthogonal planes. Structures such as the membranous labyrinth, perilymphatic compartments of the whole inner ear, vestibular end organs, vestibulocochlear- and facial-nerve were visualized using volume and surface renderings (**Figure 7**). In some specimens endolymphatic duct as well as the cochlear aqueduct were traced (**Figure 7H**). In one big human temporal bone specimen, selected features like the auditory ossicles and membranous labyrinth were segmented and visualized in Amira^®^ (**Figure 8**).

### Analysis on Tissue Volume Changes During Sample Processing

**FIGURE 9:**
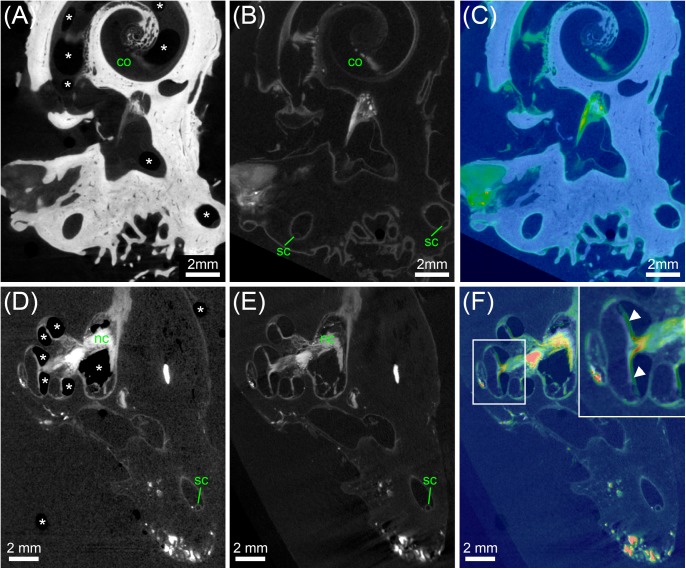
Analysis of tissue shrinkage during decalcification and transfer from PBS to 70% ethanol. **(A)** MicroCT scan of an ossified human inner ear contrasted with OsO_4_. **(B)** MicroCT scan of the same specimen shown in **(A)** after decalcification and image registration. **(C)** Colorwash overlay of ossified (gray) and decalcified (*physics* colormap) image volume after registration. The result of image registration after fine optimization suggested that specimen volume was 1.62% larger after decalcification. **(D)** MicroCT scan of a decalcified human inner ear contrasted with OsO_4_ in PBS. **(E)** MicroCT scan of the same specimen shown in **(D)** after transfer to 70% ethanol and image registration. **(F)** Colorwash overlay of decalcified sample in PBS (gray) and after transfer to 70% ethanol (*physics* colormap) after image registration. The result of image registration after fine optimization suggested that specimen volume was 0.55% larger after transfer to 70% ethanol. Legend: asterisks, air bubbles (frequently occurring if samples are in PBS); arrowheads, misalignment of some structure in cochlea based on air bubbles in the PBS scan versus liquid in the 70% ethanol scan; co, cochlea; sc, semi-circular canal.

In order to measure volume changes for different tissue preparation steps, we compared the total volume of a single specimen before and after decalcification (**Figures 9A–C**), and the total volume of a single decalcified specimen before and after transfer to 70% ethanol (**Figures 9D–F**). Pairs of image volumes were imported into Amira^®^ 6.2 and filtered using two subsequent bilateral filters. Pairs of image volumes were registered using the normalized mutual information in the *Register Images* tool, this time allowing not only rigid transformation but also isotropic and anisotropic scaling. Scaling values in the three image axes after registration were used as indicators for change in total sample volume. In addition, cross-sectional diameters of the membranous labyrinth in all semi-circular canals were measured in 4 datasets before and after transfer to 70% ethanol at 15 locations of registered image volume pairs (corresponding slices). This dual approach provided information of changes through partial dehydration to 70% ethanol in membranous structures together with the overall volume changes in the sample that is dominated by bone.

## Results

### Contrast Enhancement: Comparison of Different Compounds

In the first staining experiment on mouse specimens, I_2_KI, and PTA provided the highest contrast of soft tissue in ossified inner ears. This allows simultaneous visualization of bone and soft tissue within the same specimen acquired in one scan at 70 KeV (**Figures 1**, **2**). The procedure is ideal for screening purposes of smaller animals (mice, rats, cats, and guinea pigs, etc.) to detect variations and anomalies of gross anatomy, bone (densities) and bigger parts of the membranous labyrinth. Volumes of different compartments can be quantified across the whole inner ear. For I_2_KI, I_2_E, and OsO_4_, highest X-ray densities were achieved for the cochlear nerve, while PTA yielded highest X-ray density in the bone marrow (**Figure 2**). The generally low measured voxel intensities of OsO_4_ were most likely related to problems during fixation and/or staining in this specimen, as the second OsO_4_ specimen that was embedded in resin prior to scanning (**Figure 1F**) showed much higher staining intensities. Gastrografin^®^ did not add any detectable contrast to soft tissues of the inner ear (**Figures 1**, **2**). In the second staining experiment on mouse specimens, OsO_4_ staining was compared for two different fixation regimes comparing dual fixation with aldehydes and OsO_4_ versus fixation solely in OsO_4_. All imaged specimens provided excellent soft tissue contrast (**Figures 3A–D**). Still, differences could be seen between the two fixation strategies. The tectorial membrane was well visible only in OsO_4_-fixed specimens (**Figures 3E,G**), while the preservation of the stria vascularis was superior in the aldehyde-fixed/OsO_4_-post-fixed specimens (**Figures 3E,F**). Visibility of nerve fiber tracks was clearly superior in decalcified specimens (**Figures 3G,H**) compared to ossified specimens with higher contrast of nerve bundles and neurons in the OsO_4_-fixed specimen (**Figures 3D,H**). For a distinct presentation of nerve tissue in a decalcified specimen a single OsO_4_-fixation proved to be best.

### Dual Image Acquisition Before and After Staining

Double image acquisition in the cat specimen before and after I_2_KI-staining allowed for simultaneous imaging and visualization of temporal bone and soft tissue of the inner ear without a decalcification step (**Figure 4**). The subtraction of a bone mask from the scan of the I_2_KI-stained specimen allowed visualizing structures of the inner ear including cochlea, cochlear nerve, vestibular nerve, ampulla, and semi-circular canals in 3D (**Figure 4E**). Still, small soft tissue features such as Reissner’s membrane and the organ of Corti were not well visible in this image acquisition mode.

### Human Temporal Bone Imaging

Ossified aldehyde-fixed human temporal bones post-fixed in OsO_4_ and immersed in PBS provided good contrast of the bigger nerves within the inner ear canal (**Figure 5A**), but it was not possible to trace nerve bundles in smaller bony canals in the ossified samples. The X-ray absorption of the bone was too high to distinguish OsO_4_-stained myelinated nerve fiber bundles. Fibrous soft tissue gave even lower contrast and the delicate membranes of the endolymphatic compartment were hardly visible. After removal of mineral components with ETDA, OsO_4_ gave excellent contrast in big human inner ears and enabled to follow nerve fiber bundles along their course (**Figures 5B,E–I**). Reissner’s membrane as well as the endolymph in the vestibular system was clearly delineated from the surrounding fluid spaces (**Figures 5B,D–G**). Fibrous connective tissue gave high contrast, as visible in the vicinity of the round window niche in **Figures 5A,B**. For human temporal bones the iodine contrasting techniques of ossified specimens did not provide equal results with the equipment and settings used (**Figure 5C**), compared to smaller animal specimens (**Figure 1A**). While the vestibulocochlear nerve yielded very high contrast, membranes where not clearly visible in human specimens.

In another experiment of an ossified human cochlea we tested how enhanced image resolution can help to display soft tissue post-fixed with OsO_4_. 3 μm voxel size of an epoxy resin-embedded human cochlea gave good contrast of Reissner’s membrane, nerve fiber bundles, and fibrous tissue (**Figure 5D**). Long integration times and averaging (eight times per projection) decreased noise and enabled to trace nerve fiber bundles. The inset in **Figure 5D** emphasizes the delicate osseous spiral lamina that houses the peripheral nerve fiber bundles. The loss of myelination close to the *habenula perforata* results in lower absorption of the nerves. The outline of the organ of Corti can be distinguished and even Corti’s tunnel can be identified (**Figure 5D**). The featured characteristics of unmyelinated spiral ganglion somata in humans (Glueckert et al., 2005a; Potrusil et al., 2012) impedes recognition of single neuron bodies, so quantification of neurons may not be possible with this imaging of a human cochlea in toto. The influence of voxel resolution on smallest detectable image feature size in decalcified OsO_4_-post-fixed specimens was evaluated in **Figures 5E–G**. 15 μm voxel size is suitable to identify the membranous labyrinth and bigger nerve fiber bundles as well as fibrous tissue (**Figure 5E**). Like in most contrast enhancement methods we tested, the stria vascularis shows higher X-ray absorption that allows identification of this three cell layer thick tissue. Increasing the voxel resolution to 10 μm reduces the field of view and hence accessible specimen size, but still enables to visualize the human inner ear in toto (**Figure 5F**). Nerve fiber bundles appear much clearer and smaller individual bundles may be depicted as seen in the higher magnified inset. Increasing resolution to 5.5 μm cannot cover the whole inner ear in a single scan (**Figure 5G**) but enables to follow the peripheral nerve fibers as they fan out towards the sensory epithelium in horizontal maximum intensity projections with a high level of detail (**Figure 5H**). A maximum intensity projection in the plane perpendicular to the modiolus illustrates the contribution of different nerve bundles for the innervation of the macula sacculi, individual branches of the singular nerve and peripheral as well as central cochlear nerve fibers along the tonotopic axis (**Figure 5I**) strikingly clear.

### Resolution of Single Cells

Maximum achievable resolution with lab-based microCT setups was tested with a sub-volume scan (interior tomography) in the decalcified, solely OsO_4_-fixed mouse inner ear and compared to histological 1 μm thick semi-thin sections (**Figures 6A–D**). A voxel resolution of 0.5 μm (**Figures 6B,D**) enabled to recognize single cells. Spiral ganglion type I neurons with diameters around 10 μm can clearly be outlined as well as mesothelial cells bulging from Reissner’s membrane into the perilymphatic fluid space (**Figure 6A**). Even the acellular tectorial membrane presents as a structure well separated from the spiral limbus. A semi-thin section of a corresponding region of a different animal (**Figure 6B**) exemplifies the high level of cellular resolution possible with these scanning parameters. Nerve fibers and the stria vascularis yielded highest contrast. Latter showed some detachment, possibly as a fixation artifact because of solely OsO_4_-fixation without prior aldehyde protein fixation or mechanical stress during fixative perfusion. Horizontal views demonstrated that the high level of detail enabled to recognize single outer hair cells (**Figure 6D**). Fluid containing Nuel’s space around the lateral cell surface of outer hair cells provided enough local contrast for individual cell counts.

### Segmentation of Soft Tissue and Nerve Fiber Tracking in Human Temporal Bones

OsO_4_ post-fixation in combination with decalcification of mineral components seems to be the method of choice to selectively present myelinated nerve fibers and membranous structures together with some tissues that give higher contrast (e.g., sensory epithelia of the vestibular system and stria vascularis). Manual segmentation of the enveloping nerve branches allows to measure length, course, and diameter of these structures in 15 μm voxel sized datasets. We were able to segment the outline of the sensory epithelium in the vestibular end organs as distinct structures although we meet the limit of resolution of this cell layer. For the purpose of finite element simulation of current spread with electrical stimulation we introduced a gap region between the space of the sensory epithelium and peripheral nerve fiber bundles that give higher contrast (Handler et al., 2017). This allows setting conductivity parameters for this region that contains myelinated and unmyelinated nerve fibers (close to the basal pole of the hair cells) as well as loosely arranged fibrous tissue. The endolymphatic fluid spaces can easily be traced when manually outlined every fourth and fifth slice and interpolating in between. Doing so in all three axis of the coordinate system reduces stair-step segmentation artifacts (**Figures 7A–D**). **Figure 7E** shows the 3D representation of such a segmentation approach to visualize the five vestibular end organs with their apical poles of the sensory epithelium bathed in the membranous labyrinth and connected to their corresponding nerve branches. **Figure 7F** shows another 3D surface rendering of a specimen imaged with 15 μm voxel resolution displaying the perilymphatic space of the scala vestibuli and vestibular apparatus with vestibulocochlear nerve branches and the endolymphatic system. The course of the facial nerve was segmented in all human specimens. A volume rendering of a different specimen provided excellent views for anatomical studies (**Figure 7G**). Even very small canals can be traced with little effort, such as the endolymphatic duct, cochlear aqueduct, and the parallel accessory canal guiding the inferior cochlear vein that drains blood from the cochlea. A small blood vessel follows the course of the endolymphatic duct in a parallel way. Manual segmentation of all these structures turns out to be a laborious work but automatized segmentation strategies we tested were insufficient to provide reliable recognition of delicate structures. We fully segmented 50 human temporal bones for further assessments of anatomical variation (Johnson Chacko et al., 2018) and focused on the vestibular system.

### Big Human Temporal Bone Specimens

To address the need for a surgical planning of prototype vestibular electrode placement, bigger specimens are needed that contain not only the inner ear but also the mastoid bone and the middle ear. We avoided decalcification of the specimens since the information of the presence of bone besides soft tissue and blood vessels may be important. Direct perfusion of inner ear fluid spaces with fixative was not possible here, so we had to rely on sufficient penetration through the Eustachian tube, the inner ear canal, and pores of the fundus region that contain small holes in the modiolar trunk for guidance of the central cochlear axon bundles. We tested I_2_KI contrast enhancement but had to incubate the two specimens for 16 weeks in a low concentration I_2_KI solution to get sufficient contrast of soft tissue. Voxel resolution was raised to 25 μm voxel size for a whole specimen scan (**Figure 8A**). Contrast of soft tissue turned out to be very good and bone distinguished by higher X-ray absorption. 3D median filtering and adaption of contrast enabled us to recognize even bigger structures of the membranous labyrinth (**Figure 8A**, inset). At least for some parts of the membranous labyrinth as the region close to the oval window this technique provides sufficient contrast to segment the endolymphatic compartment without any mechanical manipulation by removing the stapes or penetrating the round window. We could display the endolymphatic space in the cochlea as well as the vestibular system. Smaller structures such as the reunion duct or endolymphatic duct cannot be covered with this technique and manual segmentation is less precise and much more time consuming due to very low contrast. Taken together, this approach may provide useful information for surgical planning. For detailed information on the membranous labyrinth smaller specimen size and techniques described above are preferable.

### Artifact Assessment: Decalcification, Air Bubbles, and Ethanol Immersion

The comparison of a single specimen before and after decalcification revealed that specimen volume was 1.62% larger after decalcification. Plausibility of registration results was carefully controlled and validated by eye. The transfer of decalcified specimens from PBS to 70% ethanol was beneficial as helped to reduce the number of air bubbles inside specimens (**Figure 9**) and increased the signal-to-noise ratio based on lower X-ray density of ethanol compared to PBS. The comparison of a single decalcified specimen before and after transfer to 70% ethanol showed that specimen volume was 0.55% larger after transfer to 70% ethanol. To assess whether delicate membranous structures suffered from volume changes we manually measured the cross-sectional diameter of the membranous labyrinth in the semi-circular canals in 4 specimens (15 locations per specimen). These 60 measurements confirmed results above, as the average measured increase in diameter of semi-circular canals was 0.56% after transfer from PBS to 70% ethanol. These measurements demonstrate astonishingly low tissue changes after chemical fixation and during decalcification and partial dehydration.

## Discussion

Non-destructive microCT imaging has been extensively used in the past years to visualize anatomical details of inner ear samples and to assess morphometric variations. So far, most of these studies focused on the bony structure and did not take into account soft tissue (Van Spaendonck et al., 2000; Wimmer et al., 2014; Schurzig et al., 2016; Ni et al., 2017; Pfaff et al., 2017). Visualization of the delicate membranous labyrinth and main nerve fibers in the inner ear is of great importance for any study to assess geometric variability to design new electrodes for electrical stimulation. Since our knowledge about micro anatomical variation of the human inner ear is still poor we focused on contrast enhancement techniques to simultaneously visualize all sensorineural components and the membranous labyrinth in animal and human temporal bones with lab-based microCT scanners. We previously studied animal and human inner ears with synchrotron radiation micro tomography (SRmicroCT), which provided excellent contrast of soft tissues when post-fixed with OsO_4_ and imaged at spatial resolutions down to the sub-cellular level (Lareida et al., 2009). Other studies have also shown high contrast of membranous structures such as Reissner’s membrane to outline peri- and endolymphatic-fluid spaces using synchrotron phase-contrast imaging (Rau et al., 2012; Elfarnawany et al., 2017). Nevertheless, accessibility and installation effort of SRmicroCT settings hampers the practicability for larger scaled high-throughput imaging studies over longer time periods. Lab-based microCTs are easier accessible and provide good image quality at reasonable costs.

### Iodine Contrast Enhancement

High X-ray attenuation by the temporal bone, which actually is the bone with highest mineral density in the human body, impedes distinct visualization of soft tissue and nerve fibers with X-ray absorption based imaging. In this work we were able to visualize soft tissues in ossified mouse inner ears very well and found I_2_KI to give highest X-ray absorption compared to other contrast enhancement methods we tested (**Figure 1A**). The membranous labyrinth was well outlined and nerve fibers as well as the stria vascularis could easily be differentiated. Double image acquisition in a differential approach with scans taken before and after contrast enhancement and subsequent image registration and subtraction of datasets (**Figure 4**) added valuable information to distinguish bone from soft tissue. This procedure may be ideal for screening purposes of smaller animals (mice, rats, cats, and guinea pigs, etc.) to detect variations and/or anomalies of gross anatomy and atrophy of the vestibulocochlear nerve in the inner ear canal. Also facial nerve abnormities may be outlined. However, tracing small nerve fiber bundles within canals inside the temporal bone was not possible and the membranous labyrinth was not always clearly visible. Moreover, we faced problems of air bubbles in the aqueous environment of the iodine solution that may have a big impact on morphometric measures (**Figure 9F**). Recently, protocols for microscopic dual-energy imaging (microDECT) have been published (Handschuh et al., 2017) that could also yield satisfying results for inner ear specimens. Discrimination of mineralized and soft tissues based on spectral properties could be achieved even without decalcification. Human specimens stained with I_2_KI (**Figure 5C**) likewise failed to provide sufficient contrast for delicate membranes or nerve fiber pathways through compact bone. The densely mineralized temporal bone in the bigger human specimens (hence increased voxel size and higher tube currents) impedes visualization of delicate membranous structures. This is in fact a problem of dynamic range, as the bone makes it necessary to image samples with higher energy spectra (70 kVp), while imaging of the membranous labyrinth would yield much better results at lower energies (45 kVp). Taken together, the low attenuation in thin-walled membranous soft tissues at high scanning energies and the high attenuation in the thick-walled temporal bone lead to comparatively low contrast in soft tissues and high image noise.

### OsO_4_ Fixation

OsO_4_-post-fixation generated only limited image contrast in scans from ossified specimens. Scanning at much higher voxel resolutions and elevated integration time can enhance image contrast (**Figure 5D**). We demonstrated this in an ossified and epoxy resin-embedded human temporal bone, but higher absorption of the mineral content of the temporal bones required heavy averaging to achieve a reasonable signal-to-noise ratio. Even 3 μm resolution with advanced microCT equipment did not fulfill our needs for a high-throughput screening of human inner ears. Removing the mineral components by techniques developed for transmission electron microscopy (TEM) (Glueckert et al., 2005b) guaranteed best ultrastructural preservation and met our demands for a distinct visualization of small nerve fiber bundles within small bony canals (**Figures 5E–I**). A good compromise for imaging the whole human inner ear in a single scan to outline relevant soft tissue structures was found at 15 μm isometric voxel resolution (**Figuress 5E**, **7A–D**, **9E**), yielding a field of view of roughly 30 mm. Imaging the same specimens at 10 μm or even 5.5 μm voxel resolution is practicable and provide fantastic details of vestibulocochlear neuronal innervation pathways (**Figures 5G–I**). Our 2K × 2K detector required more than one scan to cover a whole human inner ear specimen in toto due to smaller field of view size (roughly 20 mm FOV for 10 μm voxel size and 11 mm FOV for 5.5 μm voxel size) with our equipment. Larger field of view scanners with bigger detectors would solve this limitation.

In ossified specimens, OsO_4_ post-fixation could not provide sufficient contrast of soft tissue. Decalcification after OsO_4_-post-fixation and lower energy (45 kVp) imaging fulfilled our needs for a distinct presentation of nerve fiber bundles and the membranous labyrinth in human samples. So far, OsO_4_ was mainly used for specimen preparation in electron microscopy. OsO_4_ crosslinks unsaturated fatty acids covalently, which ensures fixation of the fatty cellular membranes. Formaldehyde and glutaraldehyde crosslink proteins but are not able to chemically fix fatty acids. Therefore OsO_4_ cannot be attributed as a classical contrast agent that somehow attaches to soft tissue structures. It is a post-fixative that provides strong additional X-ray attenuation contrast because of the heavy metal component. The fixation effect adds another big advantage over other contrast agents. Since the amount of covalently bound OsO_4_ is proportional to the amount of membranes, myelinated nerve fibers show highest contents of this heavy metal. Each myelinated central axon is about 2–3 μm in diameter surrounded by a dense arrangement of 60–83 membrane layers (Spoendlin and Schrott, 1989). The 35,000 bipolar spiral ganglion neurons in humans send out a central axon towards the brainstem and a peripheral axon to innervate the hair cells. Peripheral axons reveal only half the diameter of the central axon neurons (1–2 μm) and are ensheathed by only 20–23 myelin membranes in human (Spoendlin and Schrott, 1989). This explains the high contrast in the central nerve compared to the peripheral parts that fan out in the osseous spiral lamina and vestibular end organs. Higher incorporation of osmium ensures highest X-ray absorption in the big myelinated nerves and silhouetted them against surrounding soft tissue that contain lower membrane densities.

If information from the bone (density) and soft tissue outline is needed a dual approach with scanning the OsO_4_ contrasted specimen before and after decalcification and registering both datasets can cover these needs as we showed in **Figures 5A,B**. In the present study, we scanned 48 specimens before and after decalcification to be able to address this aspect in a future work.

Solely fixation in OsO_4_ yielded higher contrast of the myelinated nerves and is also a technique used in TEM when highest contrast of membranes is the prime goal. The sub-volume scan of the OsO_4_-fixed mouse inner ear demonstrates that current lab-based microCT scanners provide sufficient image resolution for counting neurons in the spiral ganglion and even outer hair cells. The inferior fixation of proteins and hence partial removal during washing steps may emphasize cellular membranes even better and give higher contrast in microCT as well as in TEM. The main limitation of high-resolution scanning at cellular level is the comparatively long exposure time for single projection images, which could lead to a total scanning time in the range several days for one specimen.

The OsO_4_-fixation approach combined with imaging before and after decalcification may be ideal for a rather fast characterization of anomalies of gross anatomy in smaller animals (mice, rats, cats, and guinea pigs, etc.) for giving quantitative information about hair cells and nerve fiber densities as well as bone density. Substantial loss of nerve tissue as well as degeneration of spiral ligament and stria vascularis should be able to be detected and volumes quantified with high precision across the whole inner ear. This non-destructive methodology covers several imaging modalities for a fast characterization of the inner ear in, e.g., gene knock out mice with unknown phenotype.

### Tissue Volume Changes Through Decalcification and Partial Dehydration

Techniques such as optical thin-sheet laser imaging microscopy (TSLIM) proved to provide high image resolution and contrast for inner ears of small animals like mouse or rat (Santi et al., 2009) and also human (Johnson et al., 2014) but require besides decalcification additional clearing techniques that may impact tissue morphometry more than our approach. Our assessment of tissue shrinkage caused by our chelate based decalcification and dehydration to 70% ethanol in order to get rid of air bubbles is surprisingly low. This may be attributed to the fact that we use a gentle way of decalcification with EDTA at 37°C in a neutral buffered solution and that the extended dual fixation with formaldehyde/glutaraldehyde and OsO_4_ stabilized also delicate structures enough to resist medium ethanol dehydration. Developed initially for TEM we are able to rate the excellent cellular preservation from many previous studies on human temporal bones processed for scanning and transmission EM (Glueckert et al., 2005b; Rask-Andersen et al., 2012).

### Bigger Human Temporal Bone Specimens

For very big human specimens, reduced voxel size for standard scans and enhanced processing time for decalcification did not meet our criteria for a higher throughput evaluation, so we decided for I_2_KI contrast enhancement. The purpose for surgical planning of vestibular prototype electrode insertions required image data that include both middle ear and mastoid bone, as well as soft tissues such as blood vessels, dura and muscles. This was achieved with a voxel size of 25 μm and a field of view of roughly 50 mm. With this image resolution, even the smallest skeletal muscle in the human body – the stapedial muscle – could clearly be traced along its whole length (not shown) and the membranous labyrinth in the cochlea and vestibular system could be traced partially. Contrast is much lower than in decalcified and osmium-stained tissue (**Figure 8A**) so 3D median filtering and repeated scrolling through the image stack for structure recognition is indispensable. This makes it much more difficult to safely segment the membranous labyrinth and is also applicable only for bigger structures of the endolymphatic compartment. On the other hand, this minimal invasive technique without any manipulation at the round or oval window and avoidance of dehydrating agents may be suitable for electrode insertion studies that could be combined with histological techniques to assess mechanical trauma.

### Segmentation Approaches and Morphometry

Manual segmentation of all the structures done in this study was very laborious but all methods so far tested to speeding up this process in the end failed to provide a precise tracing of delicate membranous structures. Model-based segmentation established on average image representation or multi-atlas segmentation utilizing segmented training sets may represent advanced ways to overcome many problems of manual fine structure segmentation. The latter method takes advantage of datasets of “atlases” (training images that have been previously labeled manually by an expert) (Iglesias and Sabuncu, 2015). An intensity template is registered non-rigidly to a target dataset and the resulting transformation is used to propagate the anatomical structure label of the template into the space of the target dataset (Lotjonen et al., 2010). For these methods data need to be known before and some information about anatomical variation is necessary (Fritscher et al., 2014). With our data on anatomical variation of soft tissue structures in the human inner ear we are now able to take the next step and to build up such advanced segmentation methods on basis of statistical shape models already established (unpublished data), aiming to reduce bias from manual segmentation. Combing shape and appearance model-based segmentation from microCT data with clinical CTs and high-field MRI may allow subpixel accuracy segmentation of certain structures (Demarcy et al., 2017). Information on the position and mean shape of bony and membranous labyrinth and sensory epithelia may provide relevant information for future surgery planning with augmented clinical 3D datasets.

### Outlook for Correlative Imaging of the Human Inner Ear

Our preferential preparation technique of aldehyde-fixation and OsO_4_-post-fixation offers another crucial advantage. Our specimens can further be processed for SEM, TEM, or 3D-EM methods such as serial block face SEM or focused ion beam SEM without restrictions in a correlative workflow. During the last years, microCT was used increasingly as a tool for scouting samples or for providing morphological reference for later investigation with light and/or electron microscopy (Handschuh et al., 2013; Sengle et al., 2013; Karreman et al., 2016a,b, 2017; Morales et al., 2016). For inner ear samples, microCT images could be extremely useful to detect anomalies or pathologies as we have shown previously in combinations with SRmicroCT and histology (Schmutzhard et al., 2009a,b; Glueckert et al., 2010). Subsequently, the region of interest can be precisely targeted which significantly speeds up any ultrastructural investigation. Furthermore, already established image registration protocols (Handschuh et al., 2013) would allow to put electron microscopy data at the nanometer scale in spatial 3D context of the whole inner ear geometry as provided by microCT. In future studies we plan to utilize electron microscopic analysis with exact tonotopical localization in the cochlea along with microanatomical and ultrastructural techniques to get a more comprehensive view of the fascinating and highly complex hearing and balance organ across a representative number of specimens.

## Author Contributions

RG, AR, LJC, and TP contributed to manual segmentation of temporal bones. SH, AS-F, RG, TP, AR, LJC, EP, and EB performed specimen processing, contrast enhancement, evaluation of results, and data interpretation. AS-F, RG, AR, LJC, TP, and SH developed the workflow and processed the data. SH and DS contributed to data registration. SH and DS contributed to quantifications. AS-F, EP, EB, TP, and RG contributed to temporal bone excision and processing. RG, AS-F, SH, and LJC designed the experiment, conceived and organized the study. RG and SH wrote the manuscript. SH, AS-F, RG, LJC, EP and EB proofread and corrected the manuscript.

## Conflict of Interest Statement

The authors declare that the research was conducted in the absence of any commercial or financial relationships that could be construed as a potential conflict of interest.
