# Supplementary material for: Visualization of the Membranous Labyrinth and Nerve Fiber Pathways in Human and Animal Inner Ears Using MicroCT Imaging
Source: Front Neurosci. 2018 Jul 31;12:501. doi: 10.3389/fnins.2018.00501 (PMC6079228; doi:10.3389/fnins.2018.00501)
Supplement: Supplementary file 1 [file Data_Sheet_1.pdf]

Supplementary Material

Visualization of the Membranous Labyrinth and Nerve Fibre Pathways in Human and Animal Inner Ears using MicroCT Imaging

Rudolf Glueckert, Lejo Johnson Chacko, Dominik Schmidbauer, Anneliese Schrott-Fischer*, Thomas Potrusil, Romed Hoermann, Elisabeth J. Pechriggl, Erich Brenner, Alen Reka, Stephan Handschuh

*** Correspondence:** Anneliese Schrott-Fischer: annelies.schrott@i-med.ac.at

Detailed sample preparation and tomographic image acquisition protocols

Mice

In a first experiment, we tested the properties of five different contrast agents for staining soft tissue structures of the inner ear. All five contrast agents have previously been used for either staining soft tissues for microCT imaging or clinical radiography. Lugol's iodine potassium iodine (I_2KI) and elemental iodine in absolute ethanol (I_2E) are established contrast agents and frequently used for contrast enhancement in studies on different kinds of vertebrate soft tissue samples (Metscher, 2009a; b; Gignac and Kley, 2014; Gignac et al., 2016; Handschuh et al., 2017). Gastrografin® is a water-based iodine compound of about 7% sodium amidotrizoate and 46% meglumine amidotrizoate with a long tradition of use in clinical radiography and CT (Lessman and Lilienfeld, 1959; Hong et al., 2010). Phosphotungstic acid (PTA) has been used for different vertebrate samples (Metscher, 2009a; Das Neves Borges et al., 2014) including studies on inner ear morphology in teleost fish (Schulz-Mirbach et al., 2013). Osmium tetroxide (OsO_4) is the most commonly used chemical for post-fixation in

electron microscopy tissue preparation and has already been utilized frequently as a contrast agent for microCT imaging (Johnson et al., 2006; Metscher, 2009b; Handschuh et al., 2013). In the present study, inner ears of 5 week old male C57BL/6N mice were harvested and oval and round window as well as the cochlear apex opened near the helicotrema, immediately perfused with 4% formaldehyde. The 4% formaldehyde was always freshly prepared from paraformaldehyde and frozen at -20 °C until use. Post-mortem times until fixation reached from 4-6 minutes. After 48 hours fixative immersion on an orbital shaker at 4-5 °C and thorough washes in PBS, inner ears were incubated either with I_2KI (1 % (w/v) I_2 and 2 % (w/v) KI in distilled water; 70 hrs incubation time), I_2E (1 % I_2 (w/v) in absolute ethanol, 70 hrs incubation time), Gastrographin® (Megluminamidotrizoat 660mg/ml & Natriumamidotrizoat 100 mg/ml; 120hrs incubation time), PTA (1 % (w/v) PTA in 70% ethanol; 70 hrs incubation time), or OsO_4 (1 % (w/v) OsO_4 in 0,05 M cacodylate buffer; 48 hours incubation time). After incubation, samples were washed in the contrasting agent solvent and mounted in plastic sample holders for scanning. The I_2KI sample was mounted in distilled water, the I_2 sample was mounted in absolute ethanol, the PTA sample was mounted in 70 % ethanol, and the Gastrographin® and OsO_4 samples were mounted in PBS, respectively. In addition, another OsO_4 stained sample was decalcified with EDTA as described above and dehydrated in an ascending series of ethanol. The dehydrated specimen was placed for 15 minutes in acetone, followed by an ascending series of acetone/Epon mixtures (3+1, 1+1, 1+3) and polymerized in Epon at 58 °C for 24 hours. Samples were scanned using a Scanco microCT35 (SCANCO Medical AG, Brüttisellen, CH) with an isotropic voxel resolution of 3.5 µm. The ossified I_2KI, I_2E, Gastrographin®, PTA, and OsO_4 samples were scanned at 70 kVp and 114 µA, while the decalcified and resin-embedded OsO_4 sample was scanned at 45 kVp and 177 µA.

In a second experiment we tested the specific properties of OsO_4-staining based on two different fixation regimes. 40 days old C57BL/6N mice were fixed in Karnovsky's formaldehyde-glutaraldehyde solution (mixture of 4 % formaldehyde and 5 % glutaraldehyde in 0.1 M cacodylate buffer (pH 7.3)) and subsequently post-fixed with 2 % OsO_4 in 0.05 M cacodylate buffer pH 7.3, while two samples were directly fixed in 2 % OsO4 in 0.05M cacodylate buffer pH 7.3. For each fixation regime one sample was scanned without previous decalcification and the other sample was scanned after decalcification with EDTA. Decalcification was performed at neutral pH (pH 7.2-7.4) with EDTA in PBS for 270 minutes at 37 °C with microwave support and magnetic stirring in a Milestone® Histos 5 tissue processor (Milestone® Srl, Bergamo, Italy). The four samples were washed and mounted in PBS in plastic pipette tips and scanned with an XRadia MicroXCT-400 (Carl Zeiss X-ray Microscopy, Pleasanton, CA, USA) with an isotropic voxel size of 2.19 µm. Ossified specimens were scanned at 70 kVp and 114 µA, while decalcified specimens were scanned at 45 kVp and 110 µA. In addition, a high-resolution interior tomography of the organ of Corti of the decalcified OsO4-fixed specimen was acquired at 40 kVp and 75 µA with an isotropic voxel resolution of 0.49 µm.

Cat

One young adult cat (10 months old, 4.5 kg sourced from the BRC located at Royal Victorian Eye and Ear Hospital) was deeply anesthetized (sodium pentobarbitone; Nembutal®; i.p., 60 mg/kg) and intracardially perfused with physiological saline (37 °C) containing heparin (0.1 % v/v) and sodium nitrate (0.025 % v/v), followed by 10 % neutral buffered formalin (NBF) at 4 °C. The bullae were then removed and carefully opened to access the cochlea. Round and oval windows were perforated with a microneedle and the cochleae were locally perfused with 10 % NBF. After the bony labyrinth was

excised, the specimen was kept in NBF for 24 hours and subsequently washed in PBS. A scan of the ossified bone specimen was acquired using a Scanco microCT35 at 70 kVp and 114 µA with an isotropic voxel size of 10 µm. Subsequently it was incubated in 0.5 % (w/v) I_2 and 1 % (w/v) KI in distilled water for 10 days followed by 1 % (w/v) I_2 and 2 % (w/v) KI in distilled water for 5 days. After incubation in the contrasting agent, the sample was washed and mounted in distilled water in a plastic sample holder and scanned again using a Scanco microCT35 at 70 kVp and 114 µA with an isotropic voxel size of 10 µm.

Humans

52 temporal bones from body donors were excised and fixed in Karnovsky's formaldehyde-glutaraldehyde solution for several weeks. To ensure rapid fixative penetration, oval and round windows were penetrated with a needle and the fixative gently perfused with a Pasteur pipette. Post-mortem time until fixation reached from 4-12 hours. 48 of those specimens were post-fixed in 2% OsO4 for 2 days. After thorough washes in PBS the excess bone was removed in most of these specimens with a drill to meet maximum specimen size for the microCT scanner. Samples were washed in PBS and mounted in plastic sample holders again in PBS. Scans from the ossified specimens were acquired using a Scanco microCT35 at 70 kVp and 114 µA with an isotropic voxel size of 15 µm. After scanning, specimens were decalcified in EDTA pH 7.2-7.4 for 6-8 weeks at 37°C in a Milestone® HISTOS 5 microwave tissue processor and thoroughly washed in PBS for 5 days. Subsequently, they were transferred to 50 % and 70 % ethanol 3x2 hours each, rotated on an overhead shaker (Heidolph® Reax, Heidolph Instruments, Schwabach, Germany) for 2 days and mounted in plastic sample holders again in 70 % ethanol. This procedure ensured that air bubbles present in PBS get removed. Scans from the decalcified specimens were acquired using an XRadia MicroXCT-400 at 45 kVp and 109 µA

with an isotropic voxel size of 15 µm. For evaluating the impact of voxel size on resolvable image features, one sample was also scanned at isotropic voxel sizes of 10 µm (45 kVp, 109 µA) and 5.5 µm (45 kVp, 133 µA). For comparison, the remaining four specimens were stained with Lugols I_2KI solution (1 % (w/v) I_2 and 2 % (w/v) KI in distilled water). Two specimens were drilled to the typical sample diameter and stained for 2 weeks, and they were scanned using a Scanco microCT35 at 70 kVp and 114 µA with an isotropic voxel size of 15 µm. The other two specimens were left larger in order to visualize middle ear structures together with the inner ear without any decalcification. No extra holes were made into the inner ear of these bigger bones. Fixation and contrast agents could penetrate mainly via the Eustachian tube and inner ear canal. Those two samples were incubated in low concentration I_2KI (0.2 % (w/v) I_2 and 0.4 % (w/v) KI in distilled water) for 16 weeks, and scanned using an XRadia MicroXCT-400 at 130 kVp and 60 µA with an isotropic voxel size of 25.38 µm.

One human specimen was fixed in Karnovsky's mixture and post-fixed with 1 % OsO4 in Aqua dest. Next it was dehydrated in a series of ethanol (4 x 70 % ethanol each 4 hours, 2 x 96 % ethanol 2 hours, 2x 100 % ethanol each 1 hour). Propylene oxide was used as an intermedium (2 x 1 hour), prior to incubation in a dilution of liquid epoxy resin (Spurr, 1969) and specimen was incubated in propylene oxide in equal shares overnight in closed vials. On the next day, the mixture was replaced by 100 % epoxy resin and changed three times (1 hour each in a evacuated desiccator). All steps were performed at room temperature on an orbital shaker. The specimen was polymerized at 60 °C for 48 hours without any prior decalcification. The cochlea was separated from the vestibule with a fretsaw and the block grinded to 100 x 50 x 50mm. The plastic block was imaged using an ultra-high resolution SCANCO VivaCT 100 microCT at Scanco® Medical AG headquarter (Brüttisellen, Switzerland) yielding an isotropic spatial resolution of 3 µm. The acquired image stack had a size of 5052*5052*2400

voxels. The tube voltage was set to 70 kV, and tube current was 182 μA. Signal to noise ratio was improved by averaging 8 times per frame with 1600 ms exposure time.
