# Supplementary material for: Visualization of the Membranous Labyrinth and Nerve Fiber Pathways in Human and Animal Inner Ears Using MicroCT Imaging
Source: Front Neurosci. 2018 Jul 31;12:501. doi: 10.3389/fnins.2018.00501 (PMC6079228; doi:10.3389/fnins.2018.00501)
Supplement: Supplementary file 2 [file Data_Sheet_2.pdf]

Supplementary Material

Visualization of the Membranous Labyrinth and Nerve Fibre Pathways in Human and Animal Inner Ears using MicroCT Imaging

Rudolf Glueckert, Lejo Johnson Chacko, Dominik Schmidbauer, Thomas Potrusil, Romed Hoermann, Elisabeth J. Pechriggl, Erich Brenner, Alen Reka, Anneliese Schrott-Fischer* and Stephan Handschuh

*** Correspondence:** Anneliese Schrott-Fischer: annelies.schrott@i-med.ac.at

Detailed image processing, visualization and analysis protocols

Mice

Scans from the first staining experiment (general comparison of five different contrast agents) were exported to DICOM format and imported into the commercial 3D software package Amira® 6.2 (FEI Visualization Sciences Group, Mérignac Cédex, France). Image volumes of the five ossified scans were converted to Hounsfield units (HU) using the *Arithmetic* tool. For noise reduction, two subsequent bilateral filters were applied to each volume. Virtual sections were inspected for image contrast and visibility of selected soft tissue structures of the inner ear (Figure 1). In addition, HU-standardized images allowed to measure image intensities for different tissues and thus to quantify the staining intensities for different contrast agents. Regions of interests were segmented manually using the *Segmentation Editor* for four different tissues, including cochlear nerve, spiral ganglion, bone marrow and bone (the part of the temporal bone that underlies the macula of saccule). Average intensities

including standard deviations were measured using the *Material Statistics* tool and are given in Figure 2. Scans from the second staining experiment (OsO_4 staining based on different fixation regimes) were exported to DICOM format. No further image processing or filtering was applied. Virtual sections were also inspected for image contrast and visibility of selected soft tissue structures of the inner ear (Figure 3).

Cat

The two scans from the cat specimen, which were acquired before and after I_2KI staining, were exported to DICOM format and imported into Amira® 5.5. The two image volumes were registered using the *Affine Transformation* tool (this tool is named *Register Images* in Amira version 6.0 and higher). From the unstained specimen, a binary segmentation mask was created based on threshold segmentation. Subsequently, this mask was subtracted from the I_2KI-stained scan using the *Arithmetic* tool, which allowed selective visualization of soft tissue components.

Humans

All scans from human inner ears were exported in DICOM format. For comparison of scans made from ossified and decalcified specimens at identical voxel resolutions (15 µm), two scans from the same OsO_4-stained specimen before and after decalcification were imported into Amira® 6.2. Two subsequent bilateral filters were applied to each volume. Subsequently, the two image volumes were registered using the normalized mutual information in the *Register Images* tool and corresponding

slices were extracted to assess image contrast (Figure 5 A, B). Results from this OsO_4-stained specimen were compared to representative slices from an I_2KI-stained inner ear scanned also at 15 µm voxel resolution (Figure 5 C), as well as to the OsO_4-stained specimen that was embedded in epoxy resin and scanned at 3 µm voxel resolution (Figure 5 D). In order to assess the impact of voxel resolution on smallest detectable feature size in an OsO_4-stained and decalcified specimen, three scans of the same specimen (15 µm, 10 µm, 5.5 µm voxel resolution, respectively) were imported to Amira® 6.2. Two subsequent bilateral filters were applied to each volume. The three image volumes were registered using the normalized mutual information in the *Register Images* tool, and corresponding slices were extracted to assess image resolution (Figure 5 E-G). For illustrating smallest detectable nerve fibres in the cochlea, maximum intensity projection from thick slices were made using Amira® 5.3.3. (Figure 5 H, I).
